# Postoperative Surveillance in the Postoperative vs. Intensive Care Unit for Patients Undergoing Elective Supratentorial Brain Tumor Removal: A Retrospective Observational Study

**DOI:** 10.3390/jcm14082632

**Published:** 2025-04-11

**Authors:** Stefanie Nothofer, Julia Geipel, Kathrin Aehling, Björn Sommer, Axel Rüdiger Heller, Ehab Shiban, Philipp Simon

**Affiliations:** 1 Anaesthesiology and Intensive Care, Faculty of Medicine, University of Augsburg, 86156 Augsburg, Germany; stefanie.nothofer@uni-a.de (S.N.); julia.geipel@gmx.de (J.G.); kathrin.aehling@uk-augsburg.de (K.A.); axel.heller@uk-augsburg.de (A.R.H.); 2 Department of Neurosurgery, Faculty of Medicine, University of Augsburg, 86156 Augsburg, Germany; bjoern.sommer@uk-augsburg.de; 3 Department of Neurosurgery, Carl-Thiem Hospital, 03048 Cottbus, Germany; ehab.shiban@gmx.de

**Keywords:** supratentorial craniotomy, postoperative surveillance, postoperative care unit, neurosurgery, intensive care unit

## Abstract

**Background**: Recent evidence suggests that alternative postoperative surveillance approaches for patients undergoing elective neurosurgical procedures are less resource-intensive and result in similar or fewer complications compared to high-care settings such as Intensive Care Units (ICUs). A new postoperative care protocol was established at our facility including routine PACU admission and predefined criteria for ICU admission. We aimed to demonstrate that PACU admission is a safe option for patients undergoing elective craniotomy following eventless surgery. **Methods**: This retrospective analysis included patients undergoing elective supratentorial craniotomy before and after the implementation of the new protocol. Patients with surgery between January 2020 and January 2022 and routine ICU admission were compared to patients undergoing surgery between February 2022 and March 2023 with either PACU or ICU admission based on the new protocol regarding lengths of hospital stay (LOSs), costs, and complications. **Results**: Data from a total of 405 patients, 198 patients before and 209 patients after the protocol implementation, were included. Both groups were comparable regarding demographics, American Society of Anesthesiologists (ASA) physical status classification, preexisting health conditions, and tumor entity and volume. Postoperative LOSs were significantly shorter in PACU compared to ICU patients of the same cohort (6 d vs. 11 d, *p* = 0.002). Patients with postoperative PACU transfer suffered fewer intracranial infections, surgical site infections, and pneumonia occurrences. Surgery-related complications, 30- and 90-day readmissions, and mortality rates were comparable in both groups. **Conclusions**: Postoperative PACU admission is a safe and viable option for patients undergoing elective craniotomy when selection is thorough and is associated with fewer ICU-related complications.

## 1. Introduction

Postoperative surveillance for neurosurgical patients undergoing supratentorial craniotomy has traditionally taken place in high- or medium-care settings such as the Intensive Care Unit (ICU) or Intermediate Care Unit (IMC) [1]. Both ICUs and IMCs provide the possibility of quickly identifying and treating potential postoperative complications such as bleeding, edema, and seizures, but are resource-intensive in terms of personnel, infrastructure, supplies, and technical equipment. Recent evidence suggests that most craniotomy patients neither need nor profit from standard postoperative ICU admission. Additionally, patients in the ICU are at a high risk of healthcare-acquired infections such as postoperative surgical site infections, pneumonia, and urinary tract infections [2]. Except for delayed hematoma formation, complications occur within the first six hours after surgery and are usually detected by neurological examination, which does not necessarily need to be performed in an ICU setting [3,4,5]. Most neurosurgical patients recovering from intracranial surgery do not require any intervention that needs to take place in an ICU setting [6,7]. To avoid the use of limited ICU resources and reduce costs, many institutions have developed alternative “step-down” surveillance approaches for selected craniotomy patients such as observation in the postoperative care unit (PACU). PACU offers the possibility of basic hemodynamic monitoring, pain management, and regular neurological examinations, while being more cost-efficient and less resource-intensive compared to the ICU. As the necessity of ICU admission has been increasingly questioned, even day-care approaches with early or same-day discharge have been proposed [8,9,10]. As part of this step-down approach, many facilities have implemented Enhanced Recovery After Surgery (ERAS) concepts to enhance patient recovery by improving preadmission patient factors, pre-, intra-, and postoperative care, and hospital discharge and follow-ups [11,12,13]. Studies have previously demonstrated that postoperative PACU admission as part of an ERAS concept in selected patients leads to a reduced length of hospital stay and costs while not increasing or even decreasing complication and readmission rates, as recently summarized in two systematic reviews investigating different ERAS concepts [14,15]. However, the question remains whether these positive effects of postoperative PACU admission can be achieved based solely on clinical criteria for ICU admission, but without prior implementation of an elaborate and resource-intensive ERAS concept.

Until December 2021, neurosurgical patients undergoing elective supratentorial brain tumor removal at our facility were admitted to the ICU after surgery. A new, consensus-based postoperative surveillance protocol was established in February 2022 by an interdisciplinary team of neurosurgeons and anesthesiologists. The new protocol included prolonged postoperative PACU admission until neurologically stable enough for transfer to the regular ward, rather than postoperative ICU admission. The incentive of this retrospective analysis was to investigate the safety of the postoperative PACU admission of adult neurosurgical patients undergoing elective supratentorial craniotomy without prior implementation of an ERAS protocol. We further aimed to identify preoperative risk factors associated with a higher postoperative complication rate to evaluate which patients need to be admitted to the ICU.

## 2. Materials and Methods

### 2.1. Study Design, Settings, and Patients

We conducted a retrospective analysis of adult neurosurgical patients undergoing elective craniotomy for supratentorial brain tumor removal between January 2020 and August 2023 at the University Hospital of Augsburg, Germany. Exclusion criteria were patients under the age of 18, emergent cases, supratentorial biopsies or abscesses, infratentorial brain tumors, and endoscopic procedures. Ethical approval was granted by the Ethical Committee of the Ludwig-Maximilian University, Munich, Germany, on 28 September 2023 (N° 23-0725). The study was registered in the German Clinical Trial Register (DRKS00034300) and conducted in accordance with the Declaration of Helsinki adopted by the World Medical Association in October 2013 [16].

### 2.2. New Perioperative Regimen

The new protocol was implemented into the clinical routine in February 2022. To standardize the decision-making process regarding the necessity of ICU admission, we established a guideline consisting of several pre-, peri-, and postoperative criteria (see Table 1). Of note, this change in the standard of practice was not part of the implementation of an ERAS protocol. There were no major changes in the surgical staff during the time of the new protocol implementation.

### 2.3. Patient Allocation to ICU and PACU

**Figure 1:**
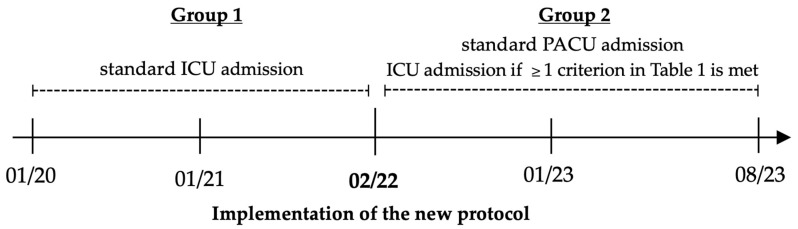
Timeline of the postoperative patient allocation process.

All patients who had surgery between January 2020 and January 2022 (Group 1), and therefore before the implementation of the new postoperative care protocol, were admitted to the ICU by default as this was the standard approach at the time. After the introduction of the new protocol in February 2022, all patients were routinely admitted to the PACU instead of the ICU. Consequently, only patients who met at least one of the criteria in Table 1 were admitted to the ICU (Group 2). The timeline of the patient allocation process is demonstrated in Figure 1.

### 2.4. Care Structure in the ICU and PACU

The surgical ICU and the PACU are managed by anesthesiologists at our maximum-care facility. The nurse-to-patient ratio is 1:2 in the ICU and 1:4 in the PACU, while the physician-to-patient ratio is 1:8 in the ICU and, during daytime, 1:16 in the PACU. Overnight, there is no physician physically present in the PACU; however, the on-call intensivist is in charge and available if needed. Unlike the ICU, our PACU is located directly next to the operating area. All patients can be monitored continuously in the PACU, and all necessary equipment for the management of complications is available at all times. This includes medications, a defibrillator, and a ventilator if non-invasive or invasive ventilation is required; however, the need for ventilation in any form is always an indication for immediate transfer to the ICU. Furthermore, both ICUs and PACUs at our institution are operated by the Department of Anesthesiology and Intensive Care Medicine; hence, both units are run by intensivists specialized in the treatment of (neuro)surgical patients and any complications that might occur postoperatively.

### 2.5. Variables and Outcomes

We collected data from all neurosurgical patients who underwent elective supratentorial brain tumor removal from January 2020 to August 2023. Patients were split into two groups depending on the time of surgery and the postoperative surveillance protocol in place at the time of surgery as previously explained. The primary outcome was defined as the postoperative length of hospital stay. Secondary outcomes include the total length of hospital stay, postoperative complications, costs, 30- and 90-day readmission rates, and 30- and 90-day all-cause mortality. Complications included the occurrence of surgical site or intracranial infections, cerebral infarction, intraoperative brain edema, postoperative bleeding, central or peripheral thromboembolisms, pneumonia, urinary tract infections, bloodstream infections, and death during the hospital stay. Readmission was defined as readmission to the neurosurgical clinic or due to surgery-associated complications resulting in hospital readmission. Preexisting health conditions (see Table 2), rheologic medication (anticoagulants and platelet aggregation inhibitors), the American Society of Anesthesiologists (ASA) physical status classification, tumor volume, localization, and entity, as well as blood loss and length of surgery, were documented. Data were extracted in a pseudonymized form from electronic patient records and anesthesia protocols. All variables were compared between group 1 and group 2 as well as between the PACU and ICU patients within group 2.

### 2.6. Statistical Analysis

Statistical analysis was carried out using SPSS statistics (IBM SPSS Statistics for Windows, Version 29.0, IBM Corp, Armonk, NY, USA) and Excel (Microsoft Excel, Version 2021, Microsoft Corporation, Redmond, Washington, DC, USA). We performed inverse probability weighting of treatment (IPWT) to assess the selection bias of being admitted to either the PACU or ICU in group 2 compared to standard ICU admission in group 1. Propensity scores were estimated by binary logistic regression, and exposure was defined as postoperative admission to either the ICU or the PACU. The final regression model included the following predictor variables: ASA physical status classification, age, neurologic, cardiac, pulmonary and nephrological preexisting health conditions, rheologic medication (including anticoagulants and platelet aggregation inhibitors), diabetes mellitus type II, tumor localization, and tumor entity (see Supplementary Table S1). The final regression model was statistically significant, x^2^ = 46.80, *p* < 0.001, and demonstrated a good fit with a Hosmer–Lemeshow test *p*-value of 0.546. A detailed description of IPWT, propensity score calculation, and the underlying regression model can be found in the Supplementary Data (Supplementary Table S1). All cases were assigned the calculated inverse probability weights, and a weighted analysis was conducted. Continuous variables were assessed for equal distribution and compared between groups by the Mann–Whitney-U test. Categorical variables were compared between groups by Pearson’s χ^2^ test. For continuous variables where more than 20% of cells had expected frequencies <5, Fisher’s exact *t*-test was used. To assess the association of admission to either PACUs or ICUs with postoperative complications, a binary logistic regression was performed for each complication separately. Data in all tables are displayed as medians [25. Quantile; 75. Quantile] for continuous variables and as absolute numbers (percentages) for categorical variables. Differences between groups were determined by the Mann–Whitney U-test for continuous variables and by Pearson’s *χ*^2^ test for categorical variables. A *p*-value of <0.05 was considered significant.

## 3. Results

### 3.1. Study Patients

We collected data from a total of 418 patients, of whom 205 were in group 1 and 213 were in group 2. We excluded 6 patients in group 1 who were admitted to PACUs instead of ICUs, leaving 199 patients in the final analysis. In group 2, 69 (32.4%) of the 213 patients were transferred to the ICU, and 142 patients (66.7%) were transferred to the PACU (Table 3). Of the 213 patients in group 2, two patients required a transfer to the ICU after initial PACU admission; these patients are addressed separately (see Supplementary Table S3). To assess the influence of the six PACU patients that we initially excluded from group 1 due to deviation from protocol, we carried out an intention-to-treat analysis regarding the primary and secondary endpoints with these patients included (see Supplementary Table S4). There was no difference when including these patients in group 1 compared to the initial per-protocol analysis, where these patients were excluded. Inverse probability weighting increased the number of patients in group 2 from 210 to 412 patients.

Patient characteristics and preexisting health conditions are displayed in Table 4. Age, sex, body mass index, and distribution of the American Society of Anesthesiologists classification did not differ significantly between group 1 and group 2 nor within ICU and PACU patients of group 2. Patients in the second group had a significantly higher incidence of diabetes mellitus type II (8.0 vs. 19.4%; *p* < 0.001). Prescription of rheologic medication, including anticoagulants and platelet aggregation inhibitors, was significantly more frequent in patients of group 1 (26.6 vs. 16.5%, *p* = 0.003) compared to group 2. Patient groups did not differ significantly regarding neurologic, pulmonary, cardiac, nephrological, or hepatic comorbidities.

### 3.2. Tumor Diagnosis and Surgical Process

The median surgical length was longer in group 1 (147 min vs. 124 min, *p* < 0.001) compared to group 2. Within all patients of group 2, the median blood loss was significantly increased in patients with postoperative ICU admission compared to the patients with routine PACU admission (300 mL vs. 500 mL, *p* < 0.001). All patients who underwent awake craniotomy in group 2 were admitted to the ICU postoperatively, as per protocol. The median tumor volume was greater in the ICU patients than in the PACU patients within group 2 (15.1 cm^3^ vs. 20.6 cm^3^, *p* = 0.026). In addition, 31% of all patients underwent surgery due to gliomas (8% with glioblastoma), 29.8% due to brain metastases, and 27.9% due to meningiomas. Notably, 48.5% of tumors were located in the frontal lobe and 18.8% were located in the temporal lobe (see Table 5).

### 3.3. Postoperative Length of Stay

There was no significant difference in the postoperative LOS between group 1 and group 2 (7 d vs. 6 d, *p* = 0.262). Of all patients with postoperative ICU admission, the patients in group 2, admitted to the ICU due to protocol criteria, had longer ICU stays than patients in group 1 with standard ICU admission (20:47 h vs. 24:48 h, *p* < 0.001). The median LOS in the PACU was significantly shorter compared to the LOS in the ICU for group 1 (16.5 h vs. 20.8 h, *p* < 0.001) as well as group 2 with ICU admission (16:45 h vs. 24:48 h, *p* < 0.001). There was no significant difference in the 30- and 90-day readmissions as well as for the 30-day mortality between group 1 and 2; however, the 90-day mortality was greater in group 1 compared to group 2 (3.5% vs. 1.0%, *p* = 0.046). All patients who died within 90 days after surgery were PACU patients of group 2. However, the cause of death was a progression of metastatic cancer that was ultimately managed in a palliative setting and not specifically related to surgical or postoperative complications in all deceased patients (see Table 6).

### 3.4. Cost Analysis

The cost analysis is based on the DRG revenue (Diagnosis-Related Group System), which is the value that a service provider in inpatient care receives from health insurance for the services rendered and can be considered as a parameter for the overall costs to the health system. It is calculated by multiplying the relative weight of resources allocated to the patient with a certain flat rate per case and covers all costs required to maintain hospital operations. The amount of reimbursement in the DRG system is based on the individual diagnosis and patient comorbidities, not on the actual treatment resources used. In this respect, it is irrelevant for reimbursement purposes whether a patient receives follow-up care in an ICU or a PACU. This makes it clear that the cost savings achieved by downgrading postoperative care to PACUs with lower staffing requirements automatically resulted in a higher net profit for all cases managed accordingly. The median total revenue was higher in group 2 compared to group 1 (EUR 15.729 vs. EUR 13.649, *p* < 0.001). When distinguishing between ICU and PACU patients of group 2, the median total revenue was significantly greater in the ICU patients than the PACU patients (EUR 16.061 vs. EUR 13.551, *p* < 0.001). The same could be observed for the nursing revenue, which was increased in the ICU patients compared to the PACU patients within group 2 (EUR 3.399 vs. EUR 2.286, *p* < 0.001) (see Table 6).

### 3.5. Complications

The most common complication in either group was postoperative bleeding, which occurred in 8.0% of patients in group 1 and 7.3% of patients in group 2. Within group 2, a significantly higher percentage of patients with ICU admission experienced postoperative bleeding compared to patients transferred to the PACU (10.8% vs. 3.5%, *p* = 0.004). More patients in group 1 suffered from pneumonia (4.5% vs. 0.7%, *p* = 0.003) as well as postoperative surgical site or intracranial infections (5.0% vs. 2.2%, *p* = 0.060). All infections were hospital-acquired infections with an onset of infection signs more than 72 h after hospital admission. Patients with intracranial infections had either meningitis or intracranial abscesses. Brain edema was more frequent in group 2 compared to group 1, presumably due to the significantly higher incidence of brain edema in the patients with ICU admission than those with PACU admission within group 2 (9.4% vs. 2.5%, *p =* 0.003). More patients in group 1 died during their hospital stay (5.5% vs. 2.4%, *p* = 0.049); however, there was no difference when distinguishing between the ICU and PACU patients in group 2. There was no difference regarding cerebral infarction, central or peripheral thromboembolisms, and urinary tract infections between group 1 and 2 (see Table 7).

Additionally, we conducted an unadjusted binary logistic regression to examine the association of postoperative transfer to either ICUs (group 1) or to PACUs/ICUs (group 2) and the likelihood of suffering from each specific complication, 30- and 90-day hospital readmissions, and all-cause mortality. The reference category was defined as ICU/PACU admission (group 2). To differentiate between patients with ICU and PACU admission in group 2, we performed the same logistic regression for group 2 with the reference category being PACU admission. Overall, patients in group 1 were 6.9 times more likely to acquire pneumonia (OR 6.908; *p* = 0.005). Patients with an ICU transfer based on ICU admission criteria (see Table 1) were 3.7 times more likely to suffer from brain edema (OR 3.693; *p* = 0.008) and 3.5 times more likely to experience postoperative bleeding (OR 3.511; *p* = 0.005) than patients with a transfer to a PACU within group 2. There was no significant association of 30- and 90-day readmissions as well as mortality and ICU or PACU admission (see Supplementary Table S2).

## 4. Discussion

The main finding of this retrospective study is that postoperative PACU admission after elective resection of supratentorial brain tumors was associated with a lower incidence of postoperative infections such as pneumonia, surgical site and intracranial infections, and reduced costs compared to ICU admission. PACU admission did not result in an increase in the postoperative length of stay, complications, readmissions, and mortality rate.

### 4.1. Current Standard of Practice and Recent Developments

There is an ongoing discussion about step-down approaches such as PACU admission, especially when it comes to patient safety, early detection and treatment of postoperative complications, readmissions, and costs. There is, without doubt, a certain reluctance when it comes to neurosurgical procedures with potentially severe complications, but the disadvantages of ICU surveillance including substantial costs, delayed mobilization, hospital-acquired infections, and prolonged LOSs must be evaluated in relation to the potential benefit [7,10]. In this study, patients of the second group were admitted to either ICUs or PACUs according to an intentional decision based on the newly implemented protocol, while the first group contained all patients regardless of whether ICU admission would have been required under the new protocol. Accordingly, differences in the primary and secondary outcomes were more apparent when comparing the ICU and PACU subgroups of the second cohort because the protocol-based classification effectively separated stable from critical patients in need of further ICU treatment. With this in mind, we demonstrated that postoperative LOSs, complications, readmissions, and mortality rates did not increase in patients with routine postoperative PACU admission even without prior implementation of a complex ERAS protocol. Furthermore, PACU patients had a significantly shorter total LOS with a median of 9 days compared to 12 days in patients with ICU admission of the same group. A decreased LOS contributes to more efficient resource utilization, reduced health care costs, and improved postoperative outcome and quality of life for the individual patient [17,18,19]. Similar results have been demonstrated before, with Florman et al. and Hoffman et al. emphasizing its relevance when optimizing postoperative patient care [9,20].

A sub-analysis of the second group showed that the incidence of postoperative bleeding and brain edema was significantly higher in patients with ICU admission compared to the PACU patients, suggesting that the interdisciplinary decision-making process regarding the necessity of ICU admission based on our implemented protocol was indeed appropriate. More ICU patients suffered from postoperative infections, such as pneumonia and surgical site as well as intracranial infections, compared to PACU patients within the second group, which are conditions that are typically associated with ICU stays [21]. This is especially important given that hospital-acquired infections—those that are not present or incubating at the time of admission and usually manifest at least 48 h after hospitalization—can result in prolonged hospitalization, severe complications, increased costs, and higher mortality rates [22,23,24,25,26].

### 4.2. Clinical and Economic Benefits of PACU Admission

By far, the greatest benefit of downgrading postoperative patient care from the ICU to the PACU is not necessarily a cost reduction, but process improvement. This means that the extremely scarce intensive care capacity can be bypassed by PACU utilization for suitable patients, which opens up the possibility of giving more patients access to urgent surgery. Key variables contributing to increased ICU costs are illness severity, length of stay, and the need for prolonged mechanical ventilation. Aside from this, patient admission itself and treatment in the first 2 days are significant cost drivers; therefore, the decision of which patients truly require ICU admission must be made carefully. We demonstrated that postoperative PACU admission after eventless supratentorial brain tumor removal led to significantly reduced total hospital as well as nursing revenue and thus contributed to a substantial reduction in inpatient hospital costs. These results align with previous cost analyses of such step-down approaches [11,14,20,27].

Routine postoperative ICU admission is a time- and resource-intensive step in the postoperative management of neurosurgical patients undergoing elective craniotomy, and its necessity has been called into question in recent years. Our results align with previous studies investigating alternative step-down approaches, such as outpatient pathways [8,28], PACU [5,9,20] or other non-ICU settings such as overnight PACU surveillance, transfer to the ward, or 4 h PACU-to-ward admission [3,7,12,27,29,30], reporting lower complication rates and comparable or even reduced lengths of postoperative and total LOS as well as readmission and mortality rates. In contrast to most other studies, we demonstrated that such beneficial effects can be replicated by basing decisions on easily accessible, clinical criteria (Table 1) with complex cases addressed through an interdisciplinary consensus between anesthesiologists and neurosurgeons.

### 4.3. Limitations

There are some limitations to this retrospective analysis. Common to all historical comparisons, there is a potential selection bias firstly between the groups, but also regarding the choice of the postoperative care level by the surgeons and anesthesiologists. All patients were treated during the global COVID-19 pandemic, which could be a significant confounding factor, particularly regarding the postoperative pneumonia rate. However, all patients required a negative SARS-CoV-2 test before undergoing any procedure, so no patient was infected at the time of surgery. Of the patients diagnosed with pneumonia postoperatively, one was infected with SARS-CoV-2, while all other patients suffered from bacterial pneumonia. Criteria such as diabetes, advanced age, high intraoperative blood loss, long surgical duration, and the type and location of the tumor have been suggested to predict the need for ICU interventions [10,31]. Such criteria might not be applicable to each individual patient, and the challenge of identifying pre- and intraoperative criteria to single out those in actual need of ICU surveillance and treatment remains. In our study, neither patient characteristics nor preexisting comorbidities were suitable variables that could predict postoperative complications, where ICU admission would be the safer choice. We believe that the reason for this is the somewhat imprecise formulation of ICU admission criteria and, potentially, even the selection of these criteria. The establishment of these predefined criteria has proven to be a challenge in the process of implementing a new postoperative care protocol, and we will need to revise and specify criteria for ICU admission.

## 5. Conclusions

The results of this study suggest that postoperative PACU is a safe and viable option for elective craniotomy patients when selected thoroughly. The implementation of a consensus-driven protocol developed by neurosurgeons and anesthesiologists has proven to be an effective measure to select patients requiring ICU admission, but the underlying criteria should be formulated precisely and specifically enough to make informed and evidence-based decisions. Furthermore, PACUs at institutions that aim to implement such step-down approaches should be operated by personnel trained and specialized in intensive care medicine to ensure safe and high-quality care and management of complications. We demonstrated that PACU admission did not increase postoperative lengths of hospital stay, readmissions, and mortality rates, and led to a decrease in ICU-associated complications as well as hospital costs.

## Figures and Tables

**Table 1:** Criteria for ICU admission according to the new postoperative care protocol. Patients who meet at least one of these criteria will be admitted to the ICU instead of routine PACU admission.

Preoperative Criteria	
Anesthesia	
Presence of patient-related risk factors based on preexisting health concerns	
Neurosurgical	
Thalamic or hypothalamic tumors	
Hormone-active pituitary gland tumors	
Skull base tumors	
Posterior falx meningiomas	
Perioperative Criteria	
Awake craniotomy	
Intraoperative complications such as brain edema or massive hemorrhage	
High risk for postoperative bleeding, dysphagia, or brain edema	
Blood loss > 1500 mL	
Duration of surgery > 240 min	
Postoperative Criteria	
Sufficient oxygenation after extubation in the operating room	
Adequate waking response and vigilance after anesthesia emergence	

**Table 2:** Documentation of preexisting health conditions.

Neurologic	Pulmonary	Cardiac/Metabolic	Hepatic	Nephrological
Epilepsy	COPD	Arterial hypertension	Chronic hepatitis B	Chronic kidney failure
Stroke/TIA	OSAS	Atrial fibrillation	Liver fibrosis	Renal cell carcinoma
Myelopathy	Lung carcinoma	Chronic heart failure	Liver cirrhosis	
Neuropathy	Lung emphysema	Coronary artery disease	Hepatomegaly	
Major TBI	Lung fibrosis	CABG/PCI		
Psychosis	Asthma bronchiale	Diabetes mellitus		
Depression	Smoking			

CABG, coronary artery bypass graft; COPD, chronic obstructive pulmonary disease; OSAS, obstructive sleep apnea syndrome; PCI, percutaneous coronary intervention; TBI: traumatic brain injury; TIA, transient ischemic attack.

**Table 3:** Initial patient population before inverse probability weighting.

**Group 1: 01/2020–01/2022**		
Total patients		205
Included in analysis	ICU admission as per protocol	199
Excluded from analysis	PACU admission ^1^	6
**Group 2: 02/2022–08/2023**		
Total patients		213
Included in analysis		211
	PACU admission as per protocol	142
	ICU admission	69
	Transfer to ICU after initial PACU admission	2

^1^ Patients in cohort 1 with postoperative PACU admission were assigned to group 2. This deviation from protocol occurred around the time of the new protocol implementation.

**Table 4:** Baseline patient demographics and preexisting health conditions.

	Group 1 (n = 199)	Group 2 (n = 412)		*p*-Value
		PACU (n = 199)	ICU (n = 213)	
**Baseline patient characteristics**				
Sex, female	106 (53.3)	214 (51.9)		0.759
		105 (52.8)	109 (51.2)	0.747
Age, years	65 [55 to 77]	64 [55 to 74]		0.379
		64 [52 to 74]	66 [55 to 75]	0.184
Body mass index (kg m^−2^)	25.6 [22.5 to 29.4]	26.6 [23.2 to 29.8]		0.139
		26.1 [23.7 to 29.7]	27.0 [22.4 to 29.8]	0.706
ASA physical status				
II	67 (33.8)	139 (33.7)		
III	120 (60.6)	259 (62.9)		
IV	11 (5.6)	14 (3.4)		
**Preexisting health conditions**				
Neurologic	52 (26.1)	133 (32.3)		0.121
		59 (29.6)	74 (34.7)	0.269
Pulmonary	56 (28.1)	125 (30.3)		0.577
		65 (32.7)	60 (28.2)	0.321
Cardiac	91 (45.7)	204 (49.5)		0.380
		90 (45.2)	114 (53.5)	0.092
Nephrological	15 (7.5)	28 (6.3)		0.743
		13 (6.5)	15 (7.1)	0.827
Hepatic	9 (4.5)	18 (4.4)		0.931
		7 (3.5)	11 (5.2)	0.414
Diabetes	16 (8.0)	80 (19.4)		**<0.001**
		32 (16.1)	48 (22.5)	0.098
Rheologic medication	53 (26.6)	68 (16.5)		**0.003**
		33 (16.6)	35 (16.5)	0.984

ASA, American Society of Anesthesiologists.

**Table 5:** Surgical details, tumor diagnosis, volume, and localization.

	Group 1 (n = 199)	Group 2 (n = 412)		*p*-Value
		PACU (n = 199)	ICU (n = 213)	
**Surgical details**				
Length of surgery [min]	147 [112 to 193]	124 [98 to 163]		**<0.001**
		125 [91 to 160]	124 [105 to 172]	0.114
Blood loss [mL]	400 [200 to 600]	400 [200 to 600]		0.629
		300 [200 to 400]	500 [300 to 700]	**<0.001**
Awake craniotomy	12 (6.0)	26 (6.3)		0.887
		0 (0.0)	26 (12.3)	**<0.001**
**Tumor diagnosis**				
Tumor volume [cm^3^]	11.5 [3.6 to 42.4]	19.0 [8.2 to 46.1]		**0.024**
		15.1 [4.9 to 38.8]	20.6 [8.2 to 54.4]	**0.026**
Tumor entity				
Meningioma	55 (27.6)	115 (28.0)		0.880
Glioma	66 (33.2)	123 (29.9)		0.519
Metastasis	58 (29.1)	124 (30.2)		0.795
Other	20 (10.1)	49 (11.9)		0.736
Tumor localization				
Frontal	114 (57.3)	220 (53.3)		
Temporal	41 (20.6)	99 (24.0)		
Parietal	28 (14.1)	62 (15.0)		
Occipital	10 (5.0)	14 (3.4)		
Other	6 (3.0)	17 (4.1)		

**Table 6:** Length of stay, readmission rates, all-cause mortality, and costs after elective supratentorial brain tumor removal.

	Group 1 (n = 199)	Group 2 (n = 412)		*p*-Value
		PACU (n = 199)	ICU (n = 213)	
**Length of stay**				
Total LOS [d]	10 [7 to 17]	11 [7 to 16]		0.416
		9 [7 to 15]	12 [9 to 19]	**<0.001**
Postoperative LOS [d]	7 [5 to 10]	6 [5 to 9]		**0.045**
		6 [4 to 7]	6 [5 to 10]	0.093
Length of stay PACU/ICU [hr]	20:47 [18:05 to 23:02]	/		**<0.001**
		16:45 [14:00 to 18:43]	24:48 [19:56 to 94:16]	**<0.001**
**Readmission and mortality rate**				
30-day readmission	37 (18.6)	59 (14.4)		0.178
		23 (11.6)	36 (17.0)	0.127
30-day mortality	6 (3.0)	5 (1.2)		0.190
		4 (2.0)	1 (0.5)	0.203
90-day readmission	27 (13.6)	55 (13.4)		0.950
		23 (11.6)	33 (15.5)	0.244
90-day mortality	7 (3.5)	4 (1.0)		**0.046**
		4 (2.0)	0 (0.0)	0.054
**Costs**				
Total revenue [EUR (€)]	13,649 [11,845 to 17,939]	15,729 [12,903 to 18,672]		**<0.001**
		13,551 [12,292 to 17,940]	16,016 [13,778 to 21,571]	**<0.001**
Nursing revenue [EUR (€)]	2280 [1520 to 4126]	2781 [1792 to 5194]		**0.003**
		2286 [1469 to 3534]	3399 [2318 to 5251]	**<0.001**

LOS: length of stay.

**Table 7:** Postoperative complications after elective supratentorial brain tumor removal.

	Group 1 (n = 199)	Group 2 (n = 412)		*p*-Value
		PACU (n = 199)	ICU (n = 213)	
Surgical site or intracranial infections	10 (5.0)	9 (2.2)		0.060
		4 (2.0)	5 (2.3)	0.548
Cerebral infarction	4 (2.0)	17 (4.1)		0.178
		8 (4.0)	9 (4.2)	0.917
Brain edema	6 (3.0)	25 (6.1)		0.106
		5 (2.5)	20 (9.4)	**0.002**
Postoperative bleeding	16 (8.0)	30 (7.3)		0.739
		7 (3.5)	23 (10.8)	**0.004**
Thromboembolism	2 (1.0)	2 (0.5)		0.600
		2 (1.0)	0 (0.0)	0.233
Pneumonia	9 (4.5)	3 (0.7)		**0.003**
		2 (1.0)	1 (0.5)	0.612
Urinary tract infection	9 (4.5)	9 (2.2)		0.108
		2 (1.0)	7 (3.3)	0.177
Death	11 (5.5)	10 (2.4)		**0.049**
		3 (1.5)	8 (3.8)	0.157

## Data Availability

The data presented in this study are available upon request from the corresponding author.

## Supplementary Materials

The following supporting information can be downloaded at https://www.mdpi.com/article/10.3390/jcm14082632/s1: Supplementary Table S1 (Binary logistic regression model for inverse probability weighting analysis), Supplementary Table S2 (Association of postoperative transfer to either ICU or PACU and postoperative complications, 30- and 90 day readmissions, and all-cause mortality), Supplementary Table S3 (PACU-to-ICU patients), and Supplementary Table S4 (Overview of outcomes with PACU patients from the first group included).

## Abbreviations

The following abbreviations are used in this manuscript:

ASA	American Society of Anesthesiologists
BMI	Body Mass Index
COVID-19	Coronavirus Disease 2019
DRG	Diagnosis-Related Group System
ERAS	Enhanced Recovery After Surgery
MDPI	Multidisciplinary Digital Publishing Institute
ICU	Intensive Care Unit
IMC	Intermediate Care Unit
IPWT	Inversed Probability Weighting of Treatment
LOS	Length of Stay
PACU	Postoperative Care Unit
SARS-CoV-2	Severe Acute Respiratory Syndrome Coronavirus 2
