# Supplementary material for: Postoperative Surveillance in the Postoperative vs. Intensive Care Unit for Patients Undergoing Elective Supratentorial Brain Tumor Removal: A Retrospective Observational Study
Source: J Clin Med. 2025 Apr 11;14(8):2632. doi: 10.3390/jcm14082632 (PMC12027877; doi:10.3390/jcm14082632)
Supplement: Supplementary file 1 [file jcm-14-02632-s001.zip › jcm-3553176-supplementary.pdf]

Supplementary Data

Detailed description of the Inverse Probability Weighing Method used for Statistical Analysis

Statistical analysis was done using SPSS statistics (IBM SPSS Statistics for Windows, Version 29.0, IBM Corp, Armonk, New York) and Excel (Microsoft Excel, Version 2021, Microsoft Corporation, Redmond, Washington, United States). We performed inverse probability weighting of treatment (IPWT) to assess the selection bias of being admitted to either PACU or ICU in group 2 compared to standard ICU admission in group 1. IPWT uses propensity scores to balance baseline patient characteristics in an exposed and an unexposed group by weighting each individual by the inverse probability of receiving the actual exposure [8]. If T is the treatment or outcome indicator (1 for ICU, 0 for PACU) and X represents the covariates, the propensity score *e(X)* is estimated as

$$\boldsymbol{e}\ (\boldsymbol{X}) = \boldsymbol{P}(\boldsymbol{T} = \mathbf{1}\ /\boldsymbol{X})$$

Propensity scores were estimated by binary logistic regression, exposure was defined as postoperative admission to either ICU or PACU. The final regression model included the following predictor variables: ASA physical status classification, neurologic, cardiac, pulmonary and nephrological preexisting health conditions, rheologic medication (including anticoagulants and platelet aggregation inhibitors) and Diabetes mellitus Type II (Table 3). Baseline demographic patient characteristics such as age, sex and BMI were excluded from the regression model because their inclusion led to a worse goodness-of-fit. The regression model was statistically significant, x^2=45.96, $p<0.001$ and demonstrated a good fit with a Hosmer-Lemeshow test p-value of 0.613. Overall percentage of accuracy in classification was 76.1%, with a sensitivity of 44.9% and a specificity of 91.9%.

After estimation of propensity scores, inverse probability weights were calculated. For patients of group 2 with postoperative transfer to the ICU, weights were calculated by the inverse of the propensity score $\frac{1}{e(X)}$. For patients of group 2 with postoperative PACU admission, the weight is the inverse of one minus the propensity score for ICU patients $\frac{1}{1-\ e(X)}$. Cook's distance was calculated to detect influential observations, but no outliers were identified (Cook's distance values < 0.52). Patients in group 1 with standard postoperative ICU admission automatically received a weight of 1. All cases were assigned the calculated weights, and a weighted analysis was conducted. Continuous variables were assessed for equal distribution and compared between groups by the Mann- Whitney- U test. Categorial variables were compared between groups by Pearson's χ^2 test. For continuous variables where more than 20% of cells had expected frequencies <5, Fisher's exact t-test was used. A p value < 0.05 was considered significant. Effect sizes were estimated by Phi coefficients for dichotomous variables.

Supplementary Table S1. Binary Logistic Regression Model for Inverse Probability Weighting Analysis.

	B	*SE*	Wald	df	*p*	OR	95% CI for OR	
							Lower	Upper
Age	0.009	0.014	0.456	1	0.499	1.009	0.984	1.037
ASA II			10.224	2	0.006			
ASA III	0.874	0.434	4.054	1	0.044	2.397	1.024	5.612
ASA IV	2.999	0.992	9.151	1	0.002	20.074	2.875	140.160
Neurologic condition	-0.328	0.432	0.575	1	0.448	0.721	0.309	1.681
Pulmonary condition	0.102	0.405	0.063	1	0.802	1.107	0.501	2.446
Cardiac condition	-0.335	0.410	0.666	1	0.415	0.715	0.320	1.599
Nephrological condition	1.303	0.672	3.766	1	0.052	3.682	0.987	13.733
Rheologic Medication	1.054	0.503	4.392	1	0.036	2.869	1.071	7.688
Diabetes Type II	-0.193	0.511	0.142	1	0.706	0.825	0.303	2.245
Localization, *frontal*			12.577	8	0.127			
Localization, *frontotemporal*	22.494	21422.099	0.000	1	0.999	587630295.4	0.000	
Localization, *frontoparietal*	1.037	0.753	1.896	1	0.169	2.820	0.645	12.332
Localization, *temporal*	-0.234	0.476	0.242	1	0.623	0.791	0.312	2.011
Localization, *temporoparietal*	-0.183	0.859	0.046	1	0.831	0.832	0.155	4.484
Localization, *parietal*	-0.383	0.621	0.381	1	0.537	0.682	0.202	2.300
Localization, *parietooccipital*	-1.036	0.882	1.379	1	0.240	0.355	0.063	2.000
Localization, *occipital*	-1.563	1.134	1.897	1	0.168	0.210	0023	1.936
Localization, *other*	2.086	0.831	6.301	1	0.012	8.054	1.580	41.062
Entity, *glioma*			7.678	3	0.053			
Entity, *meningioma*	-1.222	0.495	6.104	1	0.013	0.295	0.112	0.777
Entity, *metastasis*	-1.168	0.514	5.168	1	0.023	0.311	0.114	0.851
Entity, *other*	-0.969	0.631	2.358	1	0.125	0.380	0.110	1.307
Constant	-1.307	0.888	2.166	1	0.141	0.270		

B: regression coefficient; **SE**: standard deviation; **Wald**: Wald Chi^2 test statistics; **df**: degrees of freedom; **p**: p-value; **OR**: Odds ratio; **CI**: confidence intervals; **ASA**: American Society of Anesthesiologists physical status classification.

Binary logistic regression

To assess the association of admission to either PACU or ICU on postoperative complications, a binary logistic regression was performed for each complication separately. No covariates were included because they had no significant influence on the occurrence of complications and led to a worse goodness-of-fit quantified by the Hosmer Lemeshow Test. Associations between postoperative and total LOS and postoperative transfer to ICU or PACU were assessed by negative binomial regression analysis as both variables were right skewed and over dispersed.

Supplementary Table S2. Association of postoperative transfer to either ICU or PACU and postoperative complications, 30- and 90day readmissions and all-cause mortality. Estimation of association between postoperative transfer to either ICU (group 1) or PACU/ICU (group 2) and each complication, 30- and 90-day readmission and all-cause mortality by unadjusted binary logistic regression. Thromboembolisms were not included due to an insufficient number of cases.

	Group 1: ICU (n=199) vs Group 2: PACU/ICU (n=412)							
	Group 2: PACU (n=199) vs ICU (n=213)							
	B	*SE*	Wald	df	*p*	OR	95% CI for OR	
							Lower	Upper
Surgical site infection	-0.880	0.470	3.501	1	0.061	2.412	0.959	6.064
	-0.060	0.683	0.008	1	0.930	0.942	0.247	3.590
Cerebral infarction	-0.718	0.564	1.622	1	0.203	0.488	0.162	1.472
	0.006	0.501	0.000	1	0.991	1.006	0.377	2.685
Death	0.822	0.442	2.453	1	0.063	2.274	0.956	5.411
	-0.978	0.712	1.884	1	0.170	0.376	0.093	1.520
Brain edema	-0.751	0.462	2.641	1	0.104	0.472	0.191	1.168
	-1.306	0.494	6.992	1	0.008	0.271	0.103	0.713
Urinary tract infection	0.716	0.475	2.274	1	0.132	2.047	0.807	5.194
	-1.118	0.774	2.090	1	0.148	0.327	0.072	1.489
Pneumonia	1.933	0.690	7.857	1	0.005	6.908	1.788	26.685
	0.499	1.226	0.166	1	0.684	1.647	0.149	18.208
Postoperative bleeding	0.100	0.322	0.096	1	0.757	1.105	0.588	2.077
	-1.256	0.448	7.853	1	0.005	0.285	0.118	0.686
30-day readmission	0.306	0.230	1.771	1	0.183	1.358	0.865	2.132
	-0.461	0.287	2.576	1	0.108	0.631	0.359	1.107

30-day mortality	0.845	0.599	1.990	1	0.158	2.327	0.720	7.526
	1.055	0.968	1.189	1	0.275	2.873	0.431	19.151
90-day readmission	0.008	0.252	0.001	1	0.974	1.008	0.615	1.652
	-0.346	0.293	1.395	1	0.238	0.708	0.399	1.256
90-day death	1.244	0.620	4.030	1	0.045	3.470	1.030	11.690
	*	*	*	*	*	*	*	*

ICU: intensive care unit: **PACU**: postoperative care unit; **B**: regression coefficient; **SE**: standard error; **Wald**: Wald Chi^2 test statistics; **df**: degrees of freedom; **p**: p-value; **OR**: Odds ratio; **CI**: confidence intervals. * Not enough cases.

Converted patients

Supplementary Table S3. PACU-to-ICU

Patients
**Patient 1, 62 years, female**
Uneventful resection of supratentorial metastasis, metastatic breast cancer. Focal seizure after transfer to the ward, CCT showed bleeding into the resection cavity, patient was transferred to the ICU and was treated with levetiracetam and midazolam, no further intervention was performed.
**Patient 2, 39 years, female**
Uneventful resection of a left hemispheric meningioma, unusual high urine output was noted in the PACU. Patient was transferred to the ICU and treated with desmopressin for central diabetes insipidus.

Supplementary Table S4. Overview of outcomes with PACU patients from the first group included.

	Group 1 (n=199)	Group 2 (n=422)		p-value
		PACU (n=205)	ICU (n=217)	
Length of surgery [min]	150 [112 to 194]	125 [98 to 166]		**<0.001**
		123 [90 to 158]	124 [104 to 172]	**0.037**
Blood loss [ml]	400 [200 to 600]	400 [200 to 600]		0.698
		300 [200 to 400]	500 [300 to 700]	**<0.001**
Awake craniotomy	12 (6.0)	28 (6.6)		0.774
		0 (0.0)	28 (12.9)	**<0.001**
Tumor volume [cm³]	11.5 [3.6 to 42.4]	20.5 [7.3 vs 47.7]		**0.018**
		14.5 [5.5 to 38.8]	20.6 [8.2 to 54.4]	**0.022**
Total LOS [d]	10 [7 to 17]	11 [7 to 16]		0.587
		9 [7 to 15]	12 [8 to 16]	**<0.001**
Postoperative LOS [d]	7 [5 to 10]	6 [5 to 10]		0.262
		6 [4 to 7]	6 [5 to 11]	0.138
LOS PACU/ ICU [hr]	20:47 [18:05 to 23:02]			**<0.001**
		16:34 [13:59 to 18:42]	24:27 [19:56 to 94:16]	**<0.001**
30-day readmission	37 (18.6)	63 (14.9)		0.246
		24 (11.7)	39 (18.0)	0.071
30-day mortality	6 (3.0)	6 (1.4)		0.231
		4 (2.0)	2 (0.9)	0.438
90-day readmission	27 (13.6)	59 (14.0)		0.889
		25 (12.2)	33 (5.9)	0.359
90-day mortality	7 (3.5)	4 (0.9)		**0.044**
		4 (2.0)	0 (0.0)	0.055
Total revenue [€]	13649 [11845 to 17939]	15705 [12868 to 18672]		**<0.001**
		13551 [12255 to 17560]	16061 [13774 to 21571]	**<0.001**
Nursing revenue [€]	2280 [1520 to 4126]	2750 [1780 to 5194]		**0.004**
		2287 [1469 to 3481]	3355 [2309 to 5241]	**<0.001**
Surgical Site or intracranial infection	10 (5.0)	9 (2.1)		0.051
		4 (2.0)	5 (2.3)	0.541
Cerebral Infarction	4 (2.0)	17 (4.0)		0.194
		8 (3.9)	9 (4.1)	0.898
Brain edema	6 (3.0)	26 (6.2)		0.098
		6 (2.9)	20 (9.2)	**0.007**
Postoperative Bleeding	16 (8.0)	30 (7.1)		0.685
		7 (3.4)	24 (11.1)	**0.003**
Thromboembolism	2 (1.0)	2 (0.5)		0.597
		2 (1.0)	0 (0.0)	0.235
Pneumonia	9 (4.5)	3 (0.7)		**0.003**
		2 (1.0)	1 (0.5)	0.614
Urinary Tract Infection	9 (4.5)	9 (2.1)		0.095
		2 (1.0)	7 (3.2)	0.177
Death	11 (5.5)	10 (2.4)		**0.042**
		3 (1.5)	7 (3.2)	0.340
